# Clinical Characteristics, Genetic Basis and Healthcare Resource Utilisation and Costs in Patients with Catecholaminergic Polymorphic Ventricular Tachycardia: A Retrospective Cohort Study

**DOI:** 10.31083/j.rcm2308276

**Published:** 2022-08-05

**Authors:** Cheuk To Chung, Sharen Lee, Jiandong Zhou, Oscar Hou In Chou, Teddy Tai Loy Lee, Keith Sai Kit Leung, Kamalan Jeevaratnam, Wing Tak Wong, Tong Liu, Gary Tse

**Affiliations:** ^1^Cardiac Electrophysiology Unit, Cardiovascular Analytics Group, Laboratory of Cardiovascular Physiology, 999077 Hong Kong, China; ^2^School of Data Science, City University of Hong Kong, 999077 Hong Kong, China; ^3^Faculty of Health and Medical Sciences, University of Surrey, GU2 7XH Guildford, UK; ^4^State Key Laboratory of Agrobiotechnology (CUHK), School of Life Sciences, Chinese University of Hong Kong, 999077 Hong Kong, China; ^5^Tianjin Key Laboratory of Ionic-Molecular Function of Cardiovascular Disease, Department of Cardiology, Tianjin Institute of Cardiology, Second Hospital of Tianjin Medical University, 300211 Tianjin, China

**Keywords:** CPVT, HCRU, tachycardia, genetics

## Abstract

**Background:**

This study examined the clinical characteristics, genetic basis, healthcare utilisation and costs of catecholaminergic ventricular tachycardia (CPVT) patients from a Chinese city.

**Methods:**

This was a territory-wide retrospective cohort study of consecutive CPVT patients at public hospitals or clinics in Hong Kong. Healthcare resource utilisation for accident and emergency (A&E), inpatient and outpatient attendances were analysed over 19 years (2001–2019) followed by calculations of annualised costs (in USD).

**Results:**

Sixteen patients with a median presentation age (interquartile range (IQR) of 11 (9–14) years old) were included. Fifteen patients (93.8%) were initially symptomatic. Ten patients had both premature ventricular complexes (PVCs) and ventricular tachycardia/fibrillation (VT/VF). One patient had PVCs without VT/VF. Genetic tests were performed on 14 patients (87.5%). Eight (57.1%) tested positive for the ryanodine receptor 2 (RyR2) gene. Seven variants have been described elsewhere (c.14848G>A, c.12475C>A, c.7420A>G, c.11836G>A, c.14159T>C, c.10046C>T and c.7202G>A). c.14861C>G is a novel RyR2 variant not been reported outside this cohort. Patients were treated with beta-blockers (n = 16), amiodarone (n = 3) and verapamil (n = 2). Sympathectomy (n = 8) and implantable-cardioverter defibrillator implantation (n = 3) were performed. Over a median follow-up of 13.3 years (IQR: 8.4–18.1) years, six patients exhibited incident VT/VF. At the patient level, the median (IQR) annualised costs for A&E, inpatient and outpatient attendances were $66 (40–95), $10521 (5240–66887) and $791 (546–1105), respectively.

**Conclusions:**

All patients presented before the age of 19. The yield of genetic testing was 57%. The most expensive attendance type was inpatient stays, followed by outpatients and A&E attendances.

## 1. Introduction

Cardiac ion channelopathies predispose to the development of spontaneous ventricular tachycardia/fibrillation (VT/VF) and sudden cardiac death (SCD) [1, 2, 3, 4, 5, 6]. Of these, catecholaminergic ventricular tachycardia (CPVT) is a less prevalent condition compared to Brugada syndrome (BrS) in Asia [7, 8]. It is typically caused by mutations in either ryanodine receptor 2 (RyR2) [9], or calsequestrin 2 (CASQ2) [10, 11], but other genes such as calmodulin (CALM) have been implicated [12, 13, 14]. CPVT is usually precipitated by exercise or distress, which results in bidirectional VT, typically presenting in the first two decades of life [15]. Globally, population-based data on CPVT have mainly come from Western countries. The largest registry created by the Pediatric and Congenital Electrophysiology Society of the United States has reported the characteristics of 237 patients [16, 17]. In another multi-national study including mainly patients from France, outcomes in 101 patients were reported [18], complementing smaller registry and case series studies by the same group [19, 20]. Another study reported specifically that 21 CPVT patients carried mutations in the CALM genes [12].

By contrast, data from Asia have been relatively sparse. A multi-centre Japanese registry of 78 patients found that 94% of the cases were sporadic with only 6% of the cases being familial [21]. In a national study from Japan, it was found that 30 gene mutation carriers were found for 3 genes in 50 probands [22]. Another Japanese study reported on the findings of 29 patients [23]. To corroborate, two studies from China have reported on six patients [11] and 12 patients [24]. Moreover, few studies have studied the economic burden of CPVT patients on the healthcare systems. In this study, we investigated the clinical characteristics, genetic basis, arrhythmic outcomes, healthcare utilisation and costs of CPVT patients from Hong Kong.

## 2. Methods

### 2.1 Study Population

This study was part of a wider study on cardiac arrhythmias that was approved by The Joint Chinese University of Hong Kong-New Territories East Cluster Clinical Research Ethics Committee. The cohort included consecutive patients diagnosed with CPVT between January 1st, 1997 to December 31st, 2018 in public hospitals or clinics of Hong Kong Hospital Authority [25]. Our team has previously used this system for conducting disease-based epidemiological or clinical studies for both prevalent and rare conditions [26], including Brugada syndrome [27] and long QT syndrome [28, 29]. This cohort, including clinical characteristics and genetic basis of this cohort have already been published by our team as a preprint [30] with subsequent publication of the anonymised dataset (https://zenodo.org/record/5636292). In the original study, centralised electronic health records were reviewed for patient identification and data extraction. The diagnosis was made initially by the case physicians. They were confirmed by G.T. through the review of case notes, documented ECGs, diagnostic test results, and genetic reports in reference to the 2013 HRS/EHRA/APHRS expert consensus statement (**Supplementary Table 1**). Diagnosis of CPVT was established based on the exercise treadmill test, adrenaline challenge test, or genetic testing by the participating institution at the time of entry. In this study, only deidentified data obtained from the administrative database were analysed.

### 2.2 Clinical and Electrocardiographic Data Collection

The baseline clinical data extracted from the electronic health records include: (1) sex; (2) age of first characteristic ECG presentation and last follow-up; (3) follow-up duration; (4) family history of SCD and the specific ion channelopathy; (5) syncope manifestation and its frequency; (6) presentation of sustained VT/VF and its frequency; (7) performance of electrophysiological study (EPS), 24-hours Holter study, ion channelopathy-specific genetic testing of the *RYR2* gene, and the respective results; (8) performance of echocardiogram; (9) presence of other arrhythmias; (10) implantation of implantable cardioverter-defibrillator (ICD); (11) occurrence, cause and age of death; (12) period between the initial presentation of characteristic ECG and the first post-diagnosis VT/VF episode; (13) initial disease manifestation (asymptomatic, syncope, VT/VF). In the present study, symptoms refer to syncope or VT/VF, thus asymptomatic indicates freedom from either presentation. Other arrhythmias include sick sinus syndrome, bradycardia, atrioventricular block, atrial tachyarrhythmias, and supraventricular tachyarrhythmias. Positive EPS is defined as the induction of spontaneous VT/VF that either sustained a minimum of 30 seconds or produced hemodynamic collapse. VT/VF in the present study is defined as ventricular tachyarrhythmia at a heart rate greater than 100 beats per minute sustained for at least 30 seconds.

The following automated measurements were extracted from baseline ECGs: (1) heart rate; (2) P wave duration (PWD) and PR interval; (3) QRS duration; (4) QT and QTc interval; (5) P, QRS and T wave axis; (6) amplitude of R and S wave from leads V5 and V1 respectively; (7) presence of 1st degree atrioventricular block, defined as PR-interval greater than 200 ms; (8) presence of interventricular delay, defined as QRS-interval greater or equal to 110 ms. Baseline ECG is the documented ECG taken at or the earliest after the initial characteristic ECG presentation. All ECG parameters, except for the amplitude of R- and S-wave from leads V5 and V1 respectively, were averaged across the 12 leads.

### 2.3 Statistical Analysis

Categorical variables were compared using Fisher’s exact test and reported as total number (percentage), whilst discrete and continuous variables were compared by Kruskal-Wallis one-way ANOVA and expressed as mean ± standard deviation. The mean annual VT/VF incidence rate of each subgroup was calculated by first obtaining the patient-specific rate by dividing the total number of sustained VT/VF events by the follow-up period, then averaging the rates within the subgroup. Statistical significance was defined as *p*-value < 0.05. The difference in the duration of post-diagnosis VT/VF-free survival between the pediatric and adult subgroup is compared quantitatively by the Kaplan-Meier survival curve and compared using the log-rank test. All statistical analysis was performed using R Studio (Version: 1.3.1073, Boston, MA, USA).

### 2.4 Healthcare Utilisation and Cost Analyses

Healthcare resource utilisation for accident and emergency (A&E), inpatient and outpatient attendances were analysed over a 19-year period (2001–2019). The costs for these attendances were calculated using unit costs published by the local government and then annualised. These costs were first calculated in the local currency (Hong Kong Dollars) and converted to US Dollars.

## 3. Results

### 3.1 Baseline Clinical Characteristics, Genetics, Management and Arrhythmic Outcomes 

The clinical characteristics of this cohort have been analysed and published by our team as a preprint [30]. A total of 16 consecutive patients were included. They have a median (interquartile range) age of presentation of 11 (9 to 14 years old) and 50% were female. All patients presented at or below the age of 19 years old (Table 1). Twelve patients fulfilled at least two criteria and four patients fulfilled one criterion of the 2013 HRS/EHRA/APHRS expert consensus statement (**Supplementary Table 1**). All patients were Han Chinese. Of the whole cohort, 15 (93.8%) were initially symptomatic and five patients experienced VT/VF/SCD prior to diagnosis. Ten patients had both PVCs and VT/VF, whereas one patient had PVCs without VT/VF. In addition, 14 patients experienced initial syncope. Moreover, cardiac MRI was performed on five patients, however no abnormalities were identified. Interestingly, some patients were asymptomatic, though the documentations did not reveal if they were family members of probands. The median delay between initial presentation and diagnosis was 4.5 (Q1–Q3: 0.8–8.5) months. Genetic tests were performed on 14 patients (87.5%), of which 8 patients (57.1%) tested positive for gene mutations. All mutations involved the RyR2 gene (**Supplementary Table 2**). The c.14861C>G mutation is novel and has not been described beyond our locality. All patients were treated with beta-blockers (nadolol: n = 12, metoprolol: n = 5, atenolol: n = 3, propranolol: n = 3, sotalol: n = 2), three patients received amiodarone and two received verapamil. The median dosages of the beta blockers used are: nadolol (80 mg), metoprolol (50 mg), atenolol (50 mg), propranolol (10 mg) and sotalol (80 mg). Regarding invasive treatments, sympathectomy was performed in eight patients and implantable-cardioverter defibrillators were inserted in three patients. Over a median follow-up of 13.3 years (IQR: 8.4–18.1) years, six patients exhibited incident VT/VF.

**Table 1.** **Baseline characteristics of the study cohort**.

Variable	All CPVT patients (n = 16)	*p*-value
Clinical characteristics		
Female	8 (50.0)	0.302
Presentation age (years)	10.8 ± 4.4	0.478
Diagnosis age (years)	11.4 ± 4.4	0.489
Current age (years)	20.5 ± 6.5	0.729
Follow-up duration (months)	116.3 ± 35.9	0.904
Family history of CPVT/SCD	3 (18.8)	0.137
Initial symptomatic	15 (93.8)	0.424
Initial syncope	14 (87.5)	0.696
Initial VT/VF/SCD	5 (31.3)	0.330
PVC	11 (68.8)	0.330
VT/VF	10 (62.5)	**0.016**
VT/VF post-presentation	7 (43.8)	-
Genetic test	14 (87.5)	0.696
Positive genetic test	8 (57.1)	0.207
Exercise tolerance test	15 (93.8)	0.424
Positive exercise tolerance test	13 (92.9)	0.231
EPS	2 (12.5)	0.696
Positive EPS	2 (100)	1.000
ICD	3 (18.8)	**0.013**
Holter study	14 (87.5)	0.696
Arrhythmia in Holter study	6 (46.2)	0.053
Echocardiogram	15 (93.8)	0.182
Abnormal echocardiogram	1 (6.7)	0.464
Cardiac MRI performed	5 (31.3)	0.889
Abnormal cardiac MRI	0 (0)	1.000
EEG	8 (50)	**0.039**
Positive EEG	0 (0)	1.000
Baseline ECG characteristics		
Heart rate	81 ± 24	0.323
P-wave duration	94 ± 18	0.881
PR interval	163 ± 53	0.955
QRS interval	90 ± 25	0.757
QT interval	369 ± 59	0.874
QTc interval	424 ± 34	0.836
P axis	40 ± 35	0.338
QRS axis	67 ± 29	0.203
T axis	43 ± 45	0.919
1st degree AV block	1 (6.3)	0.182
Interventricular delay	2 (12.5)	0.551

Categorical and continuous variables were compared between groups using Fisher’s exact test or *t*-test, respectively. Bolded text indicate *p *< 0.05.

### 3.2 Healthcare Resource Utilization and Costs

For the whole cohort, the total number of attendances for A&E, inpatient and outpatient settings were 86, 145 and 1251, respectively. These corresponded to costs of $13,596, $29,775 per year and $65 per patient-year for A&E admissions, $7,019,711, $1,019,515 per year and $33,341 per patient-year for inpatient stays, and $181,485, $19,060 per year and $706 per patient-year for outpatient clinics (Table 2). At the single patient level, the median (IQR) number of attendances for A&E, inpatient and outpatient settings were 5 (3–7), 7 (5–12) and 70 (44–111), respectively. These corresponded to total costs of $711 (395–1146), $151,754 (37,679–496,022) and $10,583 (6630–14,895), and annualised costs of $66 (40–95), $10,521 (5240–66,887) and $791 (546–1105).

**Table 2.** **Healthcare utilisation and costs**.

Attendence type	Attendances	Costs ($)	Annualised costs ($/year)
Accident & Emergency	5 (3–7)	711 (395–1146)	66 (40–95)
Inpatient	7 (5–12), 232 (57–757) days	151,754 (37,679–496,022)	10,521 (5240–66,887)
Outpatient	70 (44–111)	10,583 (6630–14,895)	791 (546–1105)

Median (25th to 75th percentile) values are presented. Costs are shown in US dollars.

## 4. Discussion

This is the first territory-wide cohort study of CPVT in Hong Kong. There are several major findings for the present study: (1) All patients presented before the age of 19, (2) the yield of genetic testing was 57%, identifying RyR2 mutants, of which c.14861C>G is a novel RyR2 variant not been reported outside this cohort, (3) the most expensive attendance type was inpatient stay at $33,341, followed by outpatient clinics at $706 and A&E admissions at 65 per patient-year.

Sudden cardiac death is an important clinical problem globally, with congenital and acquired causes [31, 32, 33, 34]. Of the congenital cardiac ion channelopathies, CPVT is characterised by exercise-induced bidirectional VT. International registry studies on European and North American patients have reported that there is a malignant arrhythmic phenotype associated with this disease with significant delays between initial presentation and subsequent diagnosis of around six months [17, 35]. By contrast, the epidemiology and characteristics of studies in Asia are limited. Locally in Hong Kong, the burden of CPVT is low [36]. A previous study examined a cohort of sudden arrhythmic death syndrome in young patients, which only identified two cases of CPVT [37]. In this study, we reported the findings of 16 patients, confirming a highly arrhythmic phenotype, with 10 patients with VT/VF at presentation or on follow-up. Regarding the genetic basis, this study identified eight variants. Of these, c.14848G>A [38], c.12475C>A [39], c.7420A>G [40], c.11836G>A [41], c.14159T>C, c.10046C>T [42, 43] and c.7202G>A [44] have been reported elsewhere. By contrast, c.14861C>G is a novel RyR2 variant that gives rise to the A4954G amino acid change. This mutation affects the cytoplasmic domain of the RyR2, and is expected to produce abnormalities in calcium handling, possible diastolic calcium leak and triggered arrhythmogenesis [45]. However, functional and modelling studies are needed to determine the precise mechanisms by which this structural change can lead to the generation of electrophysiological substrates [46, 47, 48].

### Strengths and Limitations

The major strengths of the present study include (1) comprehensive linkage of electronic health records within the public system of the city, with the availability of attendances at different hospitals; (2) costs were estimated using unit costs over long follow-up periods.

Several limitations should be noted for the present study. First, the retrospective nature of the study is inherently subjected to selection and information bias. However, consultations were performed at least annually for most patients, hence the patients were closely followed-up. Secondly, the predictive value of predictors is limited by the relatively small sample size as CPVT is the rarest out of the different ion channelopathies in our city. Furthermore, the family history of patients was not documented in detail, thus it is unclear if the asymptomatic patients were simply family members of probands. In addition, further stratification of patients based on their arrhythmic presentation would be more user-friendly, such as the absence of catecholaminergic arrhythmias versus the presence of catecholamine-induced PVCs, couplets, non-sustained VT or sustained VT/VF. However, due to the relatively small sample size, the extent of patient stratification is limited. Finally, Cox regression was attempted but because of the low number of patients, significant clinical or electrocardiographic predictors for arrhythmic outcomes could not be identified and the modifier effects by anti-arrhythmic agents could not be assessed. Recent work has examined the effects of different beta blockers on arrhythmogenicity in a large cohort of CPVT patients. This should be evaluated further in our cohort in a prospective setting.

## 5. Conclusions

All CPVT patients presented before the age of 19. Clinical and electrocardiographic findings are important predictors of arrhythmic outcomes. A nonparametric machine learning survival analysis achieved high accuracy to predict the probabilities of incident VT/VF.

## Supplementary Material

Supplementary material associated with this article can be found, in the online version, at https://doi.org/10.31083/j.rcm2308276.
