# Supplementary material for: Clinical Characteristics, Genetic Basis and Healthcare Resource Utilisation and Costs in Patients with Catecholaminergic Polymorphic Ventricular Tachycardia: A Retrospective Cohort Study
Source: Rev Cardiovasc Med. 2022 Aug 5;23(8):276. doi: 10.31083/j.rcm2308276 (PMC11266943; doi:10.31083/j.rcm2308276)
Supplement: Supplementary file 1 [file 2153-8174-23-8-276-s1.docx]

Supplementary Appendix

**Supplementary Table 1.** Details on the diagnostic criteria met for individual CPVT patients. The criteria proposed by the 2013 HRS/EHRA/APHRS expert consensus statement were used:

1. CPVT is diagnosed in the presence of a structurally normal heart, normal ECG, and unexplained exercise or catecholamine-induced bidirectional VT, polymorphic ventricular premature beats or VT in individuals <40 years of age.

2. CPVT is diagnosed in patients (index case or family member) who have a pathogenic mutation.

3. CPVT is diagnosed in family members of a CPVT index case with a normal heart who manifests exercise-induced PVCs or bidirectional/polymorphic VT.

4. CPVT can be diagnosed in the presence of a structurally normal heart and coronary arteries, normal ECG, and unexplained exercise or catecholamine-induced bidirectional VT, polymorphic ventricular premature beats or VT in individuals >40 years of age.

| Case number | Criteria 1 | Criteria 2 | Criteria 3 | Criteria 4 |
| --- | --- | --- | --- | --- |
| 1 | 1 | 1 | 0 | 0 |
| 2 | 1 | 1 | 0 | 0 |
| 3 | 1 | 1 | 0 | 0 |
| 4 | 1 | 1 | 0 | 0 |
| 5 | 0 | 1 | 1 (brother, mother, maternal granduncle) | 0 |
| 6 | 1 | 1 | 1 (brother, mother – not related to case 5) | 0 |
| 7 | 1 | 1 | 0 | 0 |
| 8 | 1 | 1 | 0 | 0 |
| 9 | 1 | 0 | 0 | 0 |
| 10 | 1 | 1 | 0 | 0 |
| 11 | 0 | 1 | 1 (father, sister) | 0 |
| 12 | 1 | 1 | 0 | 0 |
| 13 | 1 | 0 | 0 | 0 |
| 14 | 1 | 1 | 0 | 0 |
| 15 | 1 | 1 | 0 | 0 |
| 16 | 1 | 1 | 0 | 0 |

Supplementary Table 2. Genetic testing in individual CPVT patients. All mutations detected were in the RyR2 gene.

| Case number | Genetic test performed | Abnormal genetic test | Genetic results | Coding effect | Novel compared to overseas studies | Reference |
| --- | --- | --- | --- | --- | --- | --- |
| 1 | 1 | 1 | c.14848G>A | E4950K | No | [1] |
| 2 | 1 | 1 | c.12475C>A | Q4159K | No | [2] |
| 3 | 1 | 0 | - | - | - | - |
| 4 | 1 | 1 | c.7420A>G | R2474G | No | [3] |
| 5 | 1 | 0 | - | - |  |  |
| 6 | 1 | 1 | c.11836G>A | G3946S | No | [4] |
| 7 | 1 | 0 | - | - | - | - |
| 8 | 1 | 1 | c.14861C>G | A4954G | Yes | - |
| 9 | 0 | - | - | - | - | - |
| 10 | 1 | 1 | c.14159T>C | L4720P | No | RCV000182842 |
| 11 | 1 | 1 | c.10046C>T | S3349L | No | [5, 6] |
| 12 | 1 | 0 | - | - | - | - |
| 13 | 0 | - | - | - | - | - |
| 14 | 1 | 0 | - | - | - | - |
| 15 | 1 | 0 | 0 | - | - | - |
| 16 | 1 | 1 | c.7202G>A | R2401H | No | [7] |

Supplementary Figures

**
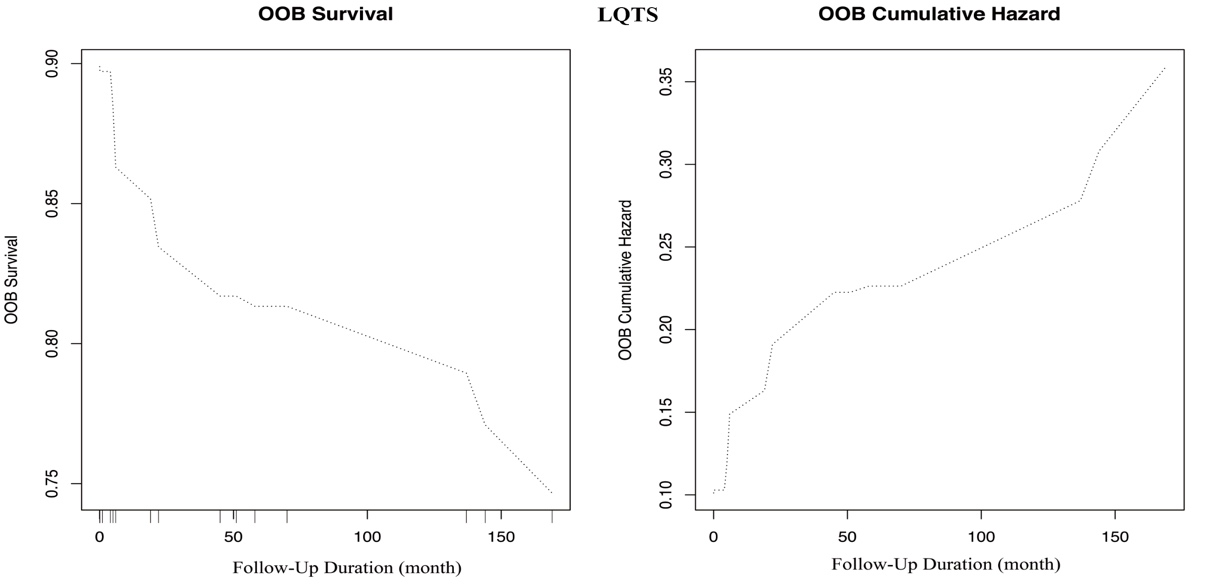
**

**
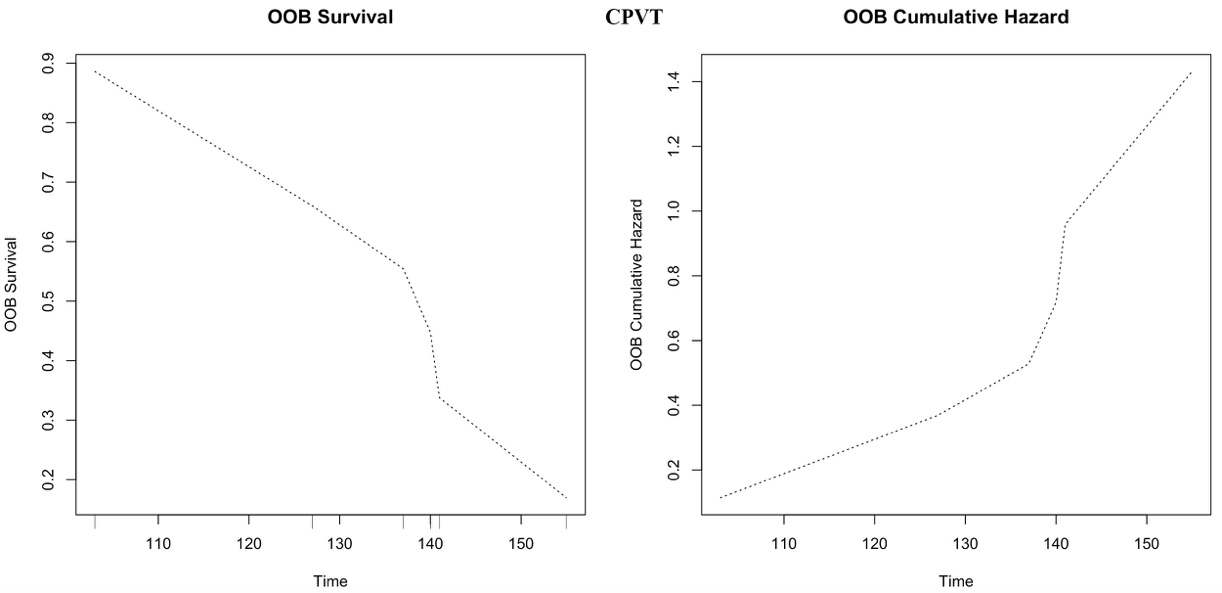
**

**Supplementary Fig. 1. Predicted OOB survivals and cumulative hazards generated by the RSF model for predicting incident VT/VF in LQTS (*a, b*) and CPVT (*c, d*).**

**
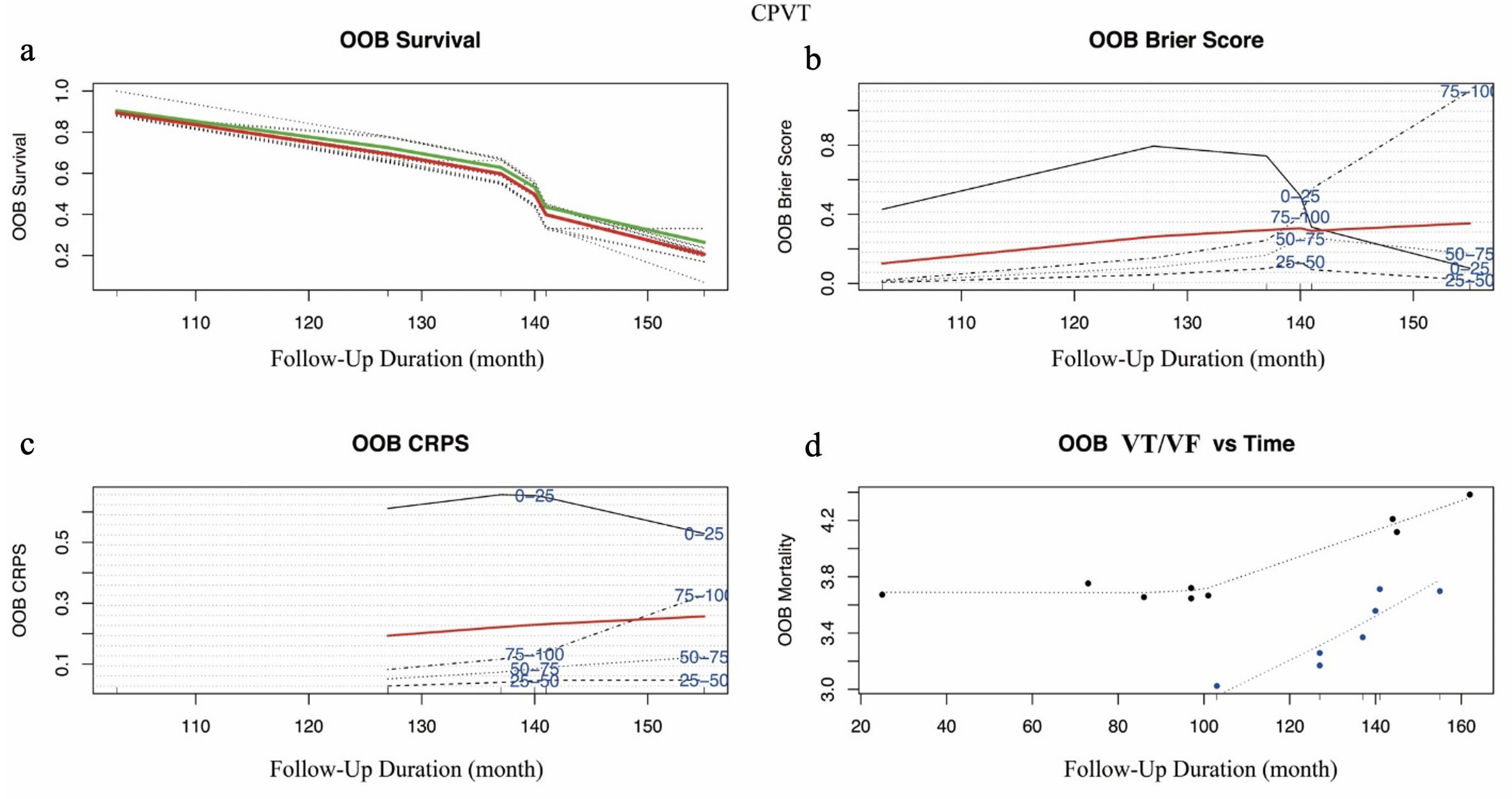
**

**Supplementary Fig. 2. Survival estimates for the CPVT cohort generated from the random survival forest (RSF) model.** The overall ensemble survival is indicated by the red line; the Nelson-Aalen estimator is given by the green line (*a*). Brier score (0=perfect, 1=poor, and 0.25=guessing) stratified by ensemble mortality based on the inverse probability of censoring weight (IPCW) method (*b)* The cohort was stratified into four groups of 0-25, 25-50, 50-75 and 75-100 percentile mortality (the overall, non-stratified, Brier score is shown by the red line). Continuous rank probability score (CRPS) given by the integrated Brier score divided by time (*c*). The plot of incident VT/VF of each LQTS patient versus observed time (*d*). Events are shown as blue points, whereas censored observations are shown as red points.

References

[1] Priori SG, Napolitano C, Memmi M, Colombi B, Drago F, Gasparini M*, et al.* Clinical and molecular characterization of patients with catecholaminergic polymorphic ventricular tachycardia. Circulation*.* 2002; 106: 69-74.

[2] Kawata H, Ohno S, Aiba T, Sakaguchi H, Miyazaki A, Sumitomo N*, et al.* Catecholaminergic Polymorphic Ventricular Tachycardia (CPVT) Associated With Ryanodine Receptor (RyR2) Gene Mutations- Long-Term Prognosis After Initiation of Medical Treatment. Circ J*.* 2016; 80: 1907-1915.

[3] Ozawa J, Ohno S, Fujii Y, Makiyama T, Suzuki H, Saitoh A*, et al.* Differential Diagnosis Between Catecholaminergic Polymorphic Ventricular Tachycardia and Long QT Syndrome Type 1- Modified Schwartz Score. Circ J*.* 2018; 82: 2269-2276.

[4] Gallegos-Cortez A, Alonso-Ortiz N, Antunez-Arguellez E, Villarreal-Molina T, Totomoch-Serra A, Iturralde-Torres P*, et al.* Catecholaminergic polymorphic ventricular tachycardia due to de novo RyR2 mutation: recreational cycling as a trigger of lethal arrhythmias. Arch Med Sci*.* 2020; 16: 466-470.

[5] Seidelmann SB, Smith E, Subrahmanyan L, Dykas D, Abou Ziki MD, Azari B*, et al.* Application of Whole Exome Sequencing in the Clinical Diagnosis and Management of Inherited Cardiovascular Diseases in Adults. Circ Cardiovasc Genet*.* 2017; 10.

[6] Christiansen SL, Hertz CL, Ferrero-Miliani L, Dahl M, Weeke PE, LuCamp*, et al.* Genetic investigation of 100 heart genes in sudden unexplained death victims in a forensic setting. European journal of human genetics : EJHG*.* 2016; 24: 1797-1802.

[7] Aizawa Y, Ueda K, Komura S, Washizuka T, Chinushi M, Inagaki N*, et al.* A novel mutation in FKBP12.6 binding region of the human cardiac ryanodine receptor gene (R2401H) in a Japanese patient with catecholaminergic polymorphic ventricular tachycardia. Int J Cardiol*.* 2005; 99: 343-345.
